# Exploring the Clinical Implication of S100A9 in Ulcerative Colitis and Its Progression to Cancer: A Journey from Inflammation to Cancer

**DOI:** 10.3390/ijms26125693

**Published:** 2025-06-13

**Authors:** Jaehwan Cheon, Sang Hyun Kim, Jaehyung Park, Tae Hoon Kim

**Affiliations:** 1 Department of Otorhinolaryngology-Head & Neck Surgery, Korea University College of Medicine, Anam-ro 145, Seongbuk-gu, Seoul 02841, Republic of Korea; 2 Department of Biomedical Science, Korea University College of Medicine, Anam-ro 145, Seongbuk-gu, Seoul 02841, Republic of Korea; 3 Department of Internal Medicine, Korea University College of Medicine, Anam-ro 145, Seongbuk-gu, Seoul 02841, Republic of Korea; 4 Mucosal Immunology Institute, Korea University College of Medicine, Anam-ro 145, Seongbuk-gu, Seoul 02841, Republic of Korea

**Keywords:** ulcerative colitis, bioinformatics, S100A9, early diagnosis, biomarker, colitis-associated cancer

## Abstract

Ulcerative colitis (UC) is a chronic inflammatory bowel disease characterized by mucosal inflammation and debilitating symptoms that considerably impair life quality. UC is particularly prevalent in younger populations, where early diagnosis remains challenging owing to nonspecific symptoms and the potential progression to colitis-associated cancer (CAC). The GSE177044 dataset, consisting of whole blood samples, was analyzed to identify differentially expressed genes, perform gene annotation, analyze key signaling pathways, and detect key hub genes in UC using protein–protein interaction networks. Multiple UC datasets composed of colonic samples were used for validation and examination of methylation and age-related gene expression patterns. Further analyses were performed to explore the association between these key hub genes and colon adenocarcinoma (COAD). We identified four key hub genes—lipocalin-2 (*LCN2*), matrix metalloproteinase-9 (*MMP9*), S100 calcium-binding protein A9 (*S100A9*), and olfactomedin-4 (*OLFM4*)—significantly up-regulated in UC, with *S100A9* showing epigenetic regulation and age-dependent expression patterns. Additionally, S100A9 was strongly associated with poor prognosis in COAD, displaying hypo-methylation and elevated expression, especially in myeloid cell types, and links to altered immune and molecular subtypes. Our findings confirmed the hypo-methylation-driven up-regulation of *LCN2*, *S100A9*, and *OLFM4* in UC, suggesting their potential as blood-based diagnostic biomarkers. Notably, S100A9 has emerged as a promising biomarker for the early diagnosis of ulcerative colitis, particularly in pediatric and adolescent patients with UC. Moreover, S100A9 holds potential as a precision target to prevent progression from UC to CAC.

## 1. Introduction

Ulcerative colitis (UC) is a persistent and relapsing subtype of inflammatory bowel disease (IBD), marked by chronic mucosal inflammation and ulceration that typically begins in the rectum and extends proximally through the colon [1,2,3]. Clinically, UC manifests with debilitating symptoms, including abdominal pain, hematochezia, and urgent defecation, all of which contribute to a significantly reduced quality of life [4,5]. The global incidence and prevalence of UC showed a steady upward trajectory by 2019, with rates in developed nations ranging between 5 and 20 cases annually per 100,000 individuals, along with an accelerating incidence in developing regions driven by shifting environmental and lifestyle factors [6,7,8]. As of 2024, UC is estimated to impact over five million individuals globally, underscoring its significant burden on public health [9].

Recent studies have increasingly characterized UC as an immune-mediated disorder with notable similarities to autoimmune diseases, in which aberrant immune responses mistakenly target the intestinal epithelium [1,10,11]. This paradigm shift in understanding is supported by accumulating evidence that genetic predispositions, environmental influences, and alterations in gut microbiota composition collectively drive the initiation and persistence of maladaptive immune responses in UC [12,13]. Despite substantial efforts to better understand and manage UC, its incidence in pediatric and adolescent populations continues to rise [14]. However, early diagnosis in these younger populations remains challenging owing to factors such as nonspecific symptoms, differences in clinical presentation as compared with that in adults, atypical manifestations, and growth delays. These complexities highlight the urgent need for improved diagnostic strategies, as current methods often lack the sensitivity and specificity required for timely and accurate detection in pediatric cases [15,16]. Moreover, recent studies have indicated an increasing incidence of patients with UC experiencing disease progression to colitis-associated cancer (CAC), which is able to ultimately advance to colorectal cancer (CRC) [17,18,19]. Consequently, identifying reliable biomarkers is essential to overcome these limitations and facilitate earlier diagnosis and intervention, particularly in younger patients at risk of UC and CRC.

Recent advances in bioinformatics have provided valuable insights into biomarker identification, facilitating the discovery of pivotal genes and functional pathways underlying the mechanisms of diverse diseases [20,21,22]. This approach represents a compelling research strategy that offers a time- and cost-efficient method for selecting disease-specific biomarkers. Moreover, it facilitates the identification of critical genes and reveals the complex molecular mechanisms underlying these key genes, thereby advancing the discovery of diagnostic targets. Building upon this foundation, we uncover the critical genes and functional pathways implicated in UC by utilizing diverse clinical datasets and robust bioinformatics approaches, with the ultimate goal of advancing our understanding of UC pathogenesis and identifying potential biomarkers for the early detection of UC and its progression to CAC.

## 2. Results

### 2.1. Discovery of Differentially Expressed Genes (DEGs) in Blood Samples from Patients with UC

**Figure 1:**
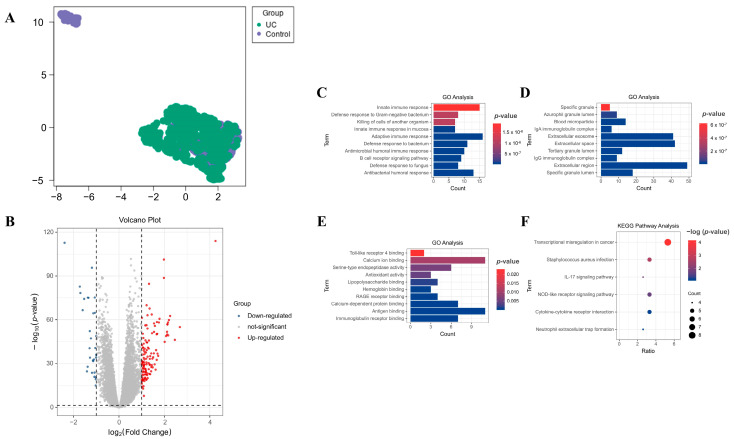
Analysis of blood-based differentially expressed genes (DEGs) and their functional annotation in patients with UC. Uniform manifold approximation and projection (UMAP) was employed to visualize individual samples within the GSE177044 dataset. Each sample is color-coded: patients with UC are represented in green, and healthy controls are indicated in purple (**A**). Volcano plot illustrating the DEG distribution patterns (**B**). In the volcano plot, red dots mark up-regulated DEGs (log_2_ fold change [log_2_FC] ≥ 1, adjusted *p*-value < 0.05), blue dots indicate down-regulated DEGs (log_2_FC ≤ −1, adjusted *p*-value < 0.05), and gray dots represent genes with no significant expression change. Gene Ontology (GO) and Kyoto Encyclopedia of Genes and Genomes (KEGG) pathway analyses were performed on these DEGs. GO analysis results are categorized by their roles in biological processes (BPs) (**C**), cellular components (CCs) (**D**), and molecular functions (MFs) (**E**). KEGG pathway analysis results highlight DEG distribution across key biochemical pathways (**F**).

A comprehensive analysis of the GSE177044 dataset using GEO2R (Figure 1A) uncovered 128 robustly up-regulated and 30 markedly down-regulated DEGs. The striking distribution of these significantly dysregulated genes is vividly illustrated in opposing volcano plots (Figure 1B).

### 2.2. Gene Ontology (GO) and Kyoto Encyclopedia of Genes and Genomes (KEGG) Pathway Analyses on DEGs

To clarify the functional significance of the identified DEGs, GO and KEGG pathway analyses were performed for all 158 DEGs. GO analysis revealed a significant enrichment of DEGs in critical biological processes (BPs), including the innate immune response, adaptive immune response, and antibacterial humoral response (Figure 1C). The analysis also revealed that DEGs were significantly enriched in cellular components (CCs), including the extracellular region, extracellular space, and extracellular exosome (Figure 1D). Moreover, in terms of molecular functions (MFs), DEGs were notably associated with Ca^2+^ binding, antigen binding, immuno-globulin receptor binding, and other related functions (Figure 1E).

KEGG pathway analysis further highlighted the enrichment of specific pathways among the DEGs, uncovering significant associations with transcriptional mis-regulation in cancer, *Staphylococcus aureus* infection, the nucleotide-binding oligomerization domain (NOD)-like receptor signaling pathway, and several other critical pathways (Figure 1F).

### 2.3. Protein–Protein Interaction (PPI) Network Construction for Hub Genes Detection

**Figure 2:**
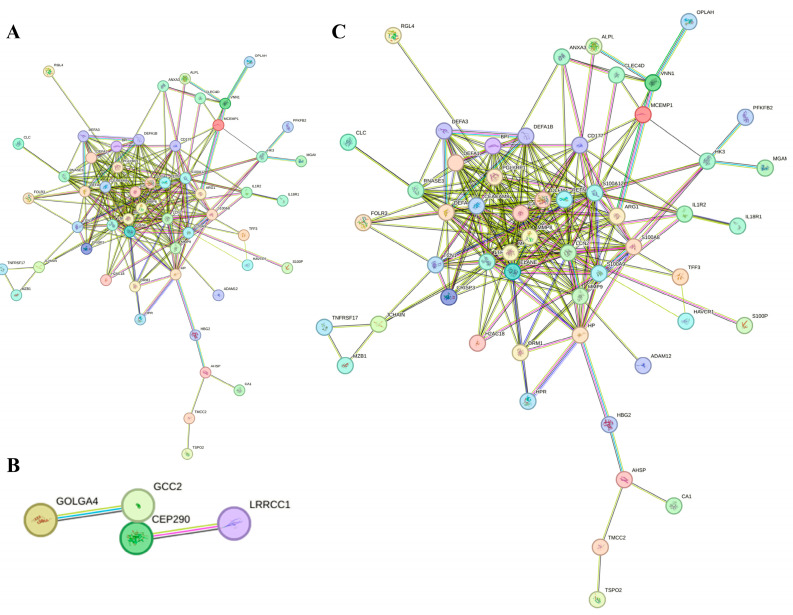
Construction of protein–protein interaction (PPI) networks. The network of up-regulated DEGs is displayed in (**A**), that of down-regulated DEGs is presented in (**B**), and the complete DEGs network is illustrated in (**C**). In each network, nodes represent genes, edges indicate protein interactions, and colors reflect the type of evidence supporting each interaction. Protein structures are enclosed within circles.

To pinpoint pivotal genes potentially involved in UC progression, we conducted an in-depth analysis of all 158 DEGs and constructed protein–protein interaction (PPI) networks using the STRING database. The resulting PPI networks detailed interactions derived from up-regulated DEGs (Figure 2A), down-regulated DEGs (Figure 2B), and the complete DEG set (Figure 2C), employing a minimum interaction score threshold of 0.4 and a highly stringent significance level of *p*-value < 1.0 × 10^−16^. The top 20 genes with the highest connectivity were identified as hub genes, each possessing a node degree of 15 or higher, out of the 158 differentially expressed genes. Notably, lipocalin-2 (*LCN2*) showed the highest connectivity, with a score of 28, followed by lactotransferrin (*LTF*) at 26, *matrix metalloproteinase* (*MMP*)*-9* at 18, S100 calcium-binding protein A9 (*S100A9*) at 16, and olfactomedin-4 (*OLFM4*) at 15.

### 2.4. Key Hub Genes Selection and Validation

**Figure 3:**
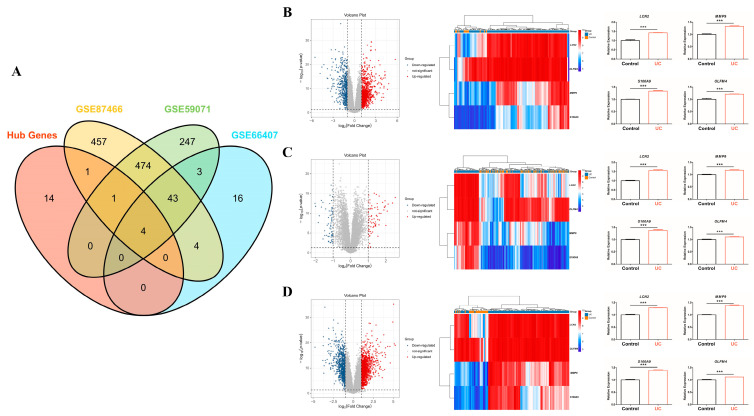
Selection of key hub genes through colonic mucosal DEGs analysis in patients with UC. By cross-referencing hub genes with gene expression profiling datasets from colonic mucosa in three datasets (GSE59071, GSE66407, and GSE87466) of patients with UC, we identified four overlapping co-hub genes as key hub genes (**A**). To validate the modified expression of these key hub genes, DEGs analysis was conducted for each gene expression dataset. All four key hub genes were consistently found within the up-regulated DEGs of each dataset (**B**–**D**). The heatmap displays hub gene expression levels with a color scale: red represents elevated expression, blue signifies reduced expression, and white denotes no notable change. Statistical significance was determined and denoted as follows: *** *p*-value < 0.001.

By cross-analyzing hub genes identified from blood samples with four gene expression profiling datasets from the colonic mucosa of patients with UC and healthy controls, we selected four co-hub genes, *LCN2*, *MMP9*, *S100A9*, and *OLFM4*, which were considered key hub genes (Figure 3A). Additionally, all the key hub genes were among the up-regulated DEGs in GSE59071 (Figure 3B), GSE66407 (Figure 3C), and GSE87466 (Figure 3D).

To evaluate the statistical robustness of key hub gene selection, we applied logistic variable selection via using logistic regression with L1 regularization (LASSO) regression with 5-fold cross-validation using a regularization parameter (C = 10). This analysis resulted in the selection of 113 genes, notably including all four key hub genes identified through network analysis: *LCN2*, *MMP9*, S100A9, and *OLFM4*, thus enabling the identification of statistically significant and highly relevant key hub genes, which improved predictive performance (Table 1).

### 2.5. Validation of Key Hub Genes Expression Through Epigenetic Factors

**Figure 4:**
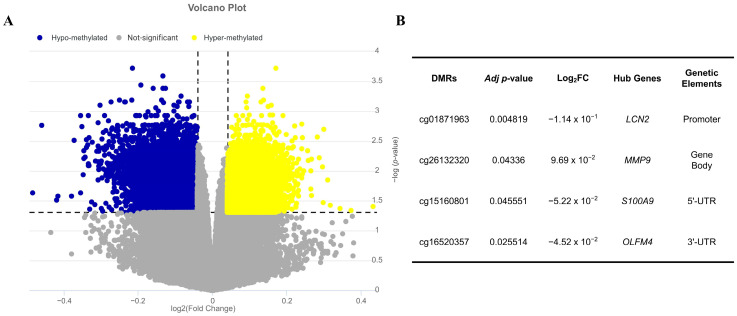
The effects of epigenetic factor on key hub gene expression in patients with UC. Epigenetic regulatory analysis affecting key hub gene expression is shown in (**A**), where the distribution of differentially methylated regions (DMRs) in the GSE32149 dataset is illustrated. Yellow dots mark significantly hyper-methylated DMRs with a log_2_FC ≥ 0.04 and a corrected *p*-value < 0.05, while blue dots represent hypo-methylated DMRs with a log_2_FC ≤ −0.04 and a corrected *p*-value < 0.05. The relationship between key hub genes and DMRs is shown in (**B**).

DNA methylation profiling of colonic mucosa samples from patients with UC and healthy controls was performed using the GS32149 dataset, identifying 2177 differentially methylated regions (DMRs), comprising 646 hyper-methylated and 1531 hypo-methylated CpG sites (Figure 4A). To evaluate the influence of methylation on hub gene expression, we analyzed the inverse correlation between DMR methylation levels and the expression of key hub genes. This analysis identified cg01871963, cg26132320, cg15160801, and cg16520357 as epigenetic regulatory elements associated with *LCN2*, *MMP9*, *S100A9*, and *OLFM4*, respectively. Notably, three DMRs (cg01871963, cg15160801, and cg16520357) were significantly hypo-methylated, whereas cg26132320 was significantly hyper-methylated (Figure 4B).

### 2.6. Age-Dependent Expression Analysis of Key Hub Genes in UC

**Figure 5:**
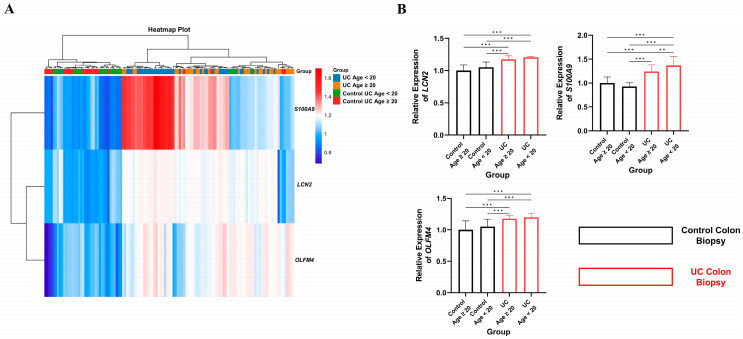
Expression levels of key hub genes in the colon of pediatric and adolescent (age < 20 years) and adult (age ≥ 20 years) patients with UC (*n* = 46 and *n* = 29, respectively). The expression levels of key hub genes, including *LCN2*, *S100A9*, and *OLFM4,* across age groups are shown in (**A**) by applying a heatmap plot. Notably, the relative expression levels of each key hub gene within each group are illustrated in (**B**). Statistical significance was determined and denoted as follows: ** *p*-value < 0.01, *** *p*-value < 0.001.

We analyzed the GSE107597 dataset to examine whether the up-regulated key hub genes *LCN2*, *S100A9*, and *OLFM4* showed differential expression patterns across age groups. Among these key hub genes, *LCN2* and *OLFM4* showed a trend in higher expression in pediatric and adolescent groups compared to adults, whereas *S100A9* exhibited markedly elevated expression in the colonic tissue (Figure 5A,B).

### 2.7. Analysis of the Effects of Key Hub Gene Expression in Cancer

**Figure 6:**
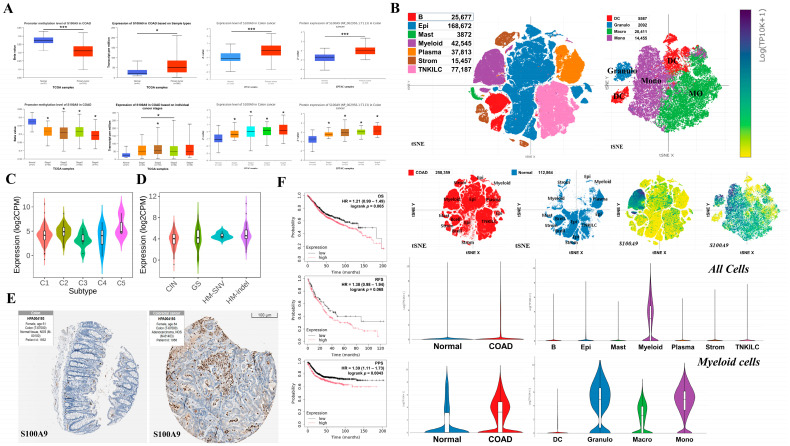
Influence of *S100A9*, a key hub gene in UC, on colorectal cancer (CRC) was examined. Analysis of the colon adenocarcinoma (COAD) dataset from *The Cancer Genome Atlas* (TCGA) revealed a marked reduction in DNA methylation levels at the *S100A9* promoter in primary tumor tissues (*n* = 313) relative to normal controls (*n* = 37). Similarly, the expression levels of *S100A9* mRNA (*n* = 286), protein (*n* = 97), and phosphorylated protein (*n* = 97) were significantly higher in tumor tissues than in normal controls. Although no significant variation in *S100A9* expression was observed across tumor stages, all stages exhibited significant differences in the protein level when compared to that in normal tissues (**A**). Analysis of the single-cell RNA sequencing (scRNA-seq) dataset for COAD demonstrated that *S100A9* expression is up-regulated across all cell types. Notably, its expression was enriched in myeloid cells, including granulocytes (granulo), monocytes (mono), macrophages (MØ), and dendritic cells (DC), and significantly elevated in COAD tumor samples (*n* = 62) compared to that in normal controls (*n* = 36) (**B**). Immune subtype analysis of *S100A9* in COAD revealed a strong association with transforming growth factor-β (TGF-β) and interferon-γ (IFN-γ)-dominant immune cell types, particularly within the C1 to C5 immune subtypes (C1: wound healing, C2: IFN-γ-dominant, C3: inflammatory, C4: lymphocyte-depleted, C5: TGF-β-dominant) (*n* = 441) (**C**). Molecular subtype analysis revealed a strong correlation between *S100A9* and hyper-mutated single nucleotide variants (HM-SNV) as well as hyper-mutated insertions/deletions (HM-indel), rather than chromosomal instability (CIN) or genome stability (GS) (*n* = 341) (**D**). Immuno-histochemistry data from the *Human Protein Atlas* (HPA) database showed more intense staining of S100A9 in COAD tumor tissues than in normal tissues (**E**). Survival analysis indicated that higher *S100A9* expression in patients with COAD is closely associated with an increased hazard ratio (HR) for overall survival (OS) (*n* = 1061), recurrence-free survival (RFS) (*n* = 1336), and PPS rates (*n* = 331) (**F**). Statistical significance was determined and denoted as follows: * *p*-value < 0.05, *** *p*-value < 0.001.

We conducted various analyses to investigate the impact of *S100A9*, a key hub gene in UC, on CRC. Consistent with our findings in UC, the promoter of *S100A9* was significantly hypo-methylated in primary tumors of CRC compared to its methylation levels in normal tissues. Furthermore, both mRNA and protein levels were significantly up-regulated compared with those in normal tissues. Moreover, phosphorylated-S100A9 (p-S100A9) was markedly elevated in primary tumors of CRC compared to its levels in normal tissues. Notably, while methylation, mRNA, protein, and phosphorylated protein levels of S100A9 did not show significant differences across cancer stages in patients with colon adenocarcinoma (COAD), all stages exhibited significant differences compared to those in normal tissues (Figure 6A). Our single-cell RNA sequencing (scRNA-seq) analysis of COAD revealed that *S100A9* expression was elevated across all cell types compared to that in normal controls, with predominant expression in myeloid cell types. Furthermore, within the myeloid cell population, *S100A9* showed notably increased expression compared to that in the controls, particularly in granulocytes, monocytes, and macrophages (MØ) (Figure 6B). Concerning immune subtypes, *S100A9* expression was predominantly elevated in cell types characterized by transforming growth factor-β (TGF-β) (C5) and interferon-γ (IFN-γ) (C2) dominance (*p*-value = 1.54 × 10^−4^) (Figure 6C). In terms of molecular subtypes, *S100A9* showed strong associations with hyper-mutated single nucleotide variants (HM-SNV) and hyper-mutated insertions/deletions (HM-indel) (*p*-value = 1.93 × 10^−3^) (Figure 6D). Consistent with the cell-level analysis, tissue staining analysis based on immuno-histochemistry (IHC) also demonstrated that S100A9 expression in the tumors of patients with COAD was elevated compared to that in normal controls (Figure 6E). Analysis of the effects of S100A9 expression on COAD revealed that higher *S100A9* expression correlated with poor prognosis. Notably, the post-progression survival (PPS) rate demonstrated a significantly greater difference (*p*-value = 0.0043 and hazard ratio [HR] = 1.39), whereas the overall survival (OS) (*p*-value = 0.066 and HR = 1.21) and recurrence-free survival (RFS) rates (*p*-value = 0.068 and HR = 1.38) were not significant (Figure 6F).

## 3. Discussion

UC, along with Crohn’s disease (CD), is a major form of IBD, with UC generally demonstrating a higher remission rate than CD [23]. Despite numerous studies conducted on UC, particularly regarding its high remission rate, its precise and comprehensive pathophysiology remains unclear, presenting considerable challenges for early diagnosis, especially in pediatric and adolescent populations [24,25,26]. Among various biological fluids, blood is a representative sample for diagnostic purposes, with a relatively noninvasive collection procedure. Thus, we aimed to identify diagnostic biomarkers of UC using bioinformatic approaches by employing the GSE177044 dataset, which comprises whole blood samples from patients with UC (*n* = 481) and healthy controls (*n* = 311), with the goal of facilitating early detection of UC.

In this present study, we conducted GO and KEGG analyses of DEGs identified in the blood of patients with UC to better elucidate the underlying pathological mechanisms of UC. GO analysis focusing on BPs revealed significant enrichment in innate immune responses, particularly the defense response to Gram-negative bacteria, as well as adaptive immune responses, with a notable emphasis on factors related to humoral immunity (Figure 1C). Consistent with this, recent studies have highlighted the influence of specific Gram-negative bacteria, including *Escherichia coli* [27], *Fusobacterium nucleatum* [28], and *Campylobacter concisus* [29], in modulating UC pathogenesis. Furthermore, growing evidence has linked humoral immune responses to UC exacerbation [30,31,32]. Notable studies have demonstrated that while colonic B-cells primarily produce IgA during homeostasis, inflammation triggers a class switch for IgG production. This switch facilitates opsonization through Fcγ-receptors on monocytes and MØ, which subsequently promotes IL-1β secretion through the engagement of the NLRP3 (nucleotide-binding domain, leucine-rich containing family, pyrin-domain containing 3) inflammasome signaling pathway, driving T-cell polarization toward TH17 differentiation in the colon [30,33]. These observations are consistent with our GO analysis findings related to CCs, where IgA and IgG immuno-globulin complexes, along with extracellular regions and spaces, were significantly enriched (Figure 1D), as were MFs, which showed enrichment in immuno-globulin receptor binding (Figure 1E). These findings support the notion that UC possesses autoimmune characteristics beyond those of a typical IBD, indicating that dysregulated immune responses targeting normal tissues may contribute to disease progression [34,35,36].

Additionally, these results were consistent with the results of our KEGG analysis, which revealed enriched cytokine–cytokine receptor interaction, NOD-like receptor, and the IL-17 signaling pathway (Figure 1F). Recent advances in immunological research have highlighted that those innate immune interactions within the colonic lamina propria—particularly among epithelial cells, dendritic cells (DCs), and MØ—are able to drive adaptive immune responses by polarizing CD4^+^ helper T-cells into various subsets, such as T_H_2 and T_H_9 [37,38]. Notably, polarization toward T_H_17 results in the secretion of IL-17A by T-cells, which subsequently promotes eosinophil infiltration [39], thereby further amplifying innate immune response activation. This aligns with previous studies indicating that IL-17 release from peripheral blood mononuclear cells (PBMCs) is elevated in patients with UC. Furthermore, the T_H_17 subset and IL-17-mediated response were found to be expanded at the single-cell level in patients with UC compared to the response in healthy controls [40,41]. Notably, Fesneau et al. reported that an aberrant T_H_17 subset is able to initiate spontaneous transformation of the epithelium, contributing to its tumorigenic potential through the production of IL-17 [42]. Our KEGG pathway analysis, which indicates enrichment in the “transcriptional mis-regulation in cancer” pathway, may be linked to CAC. For instance, Eaden et al. documented that the incidence of cancer in patients with UC was 2.1% after 5 years, increased to 8.5% after 10 years, and reached 17.8% after 20 years of disease progression [43]. Thus, discovering biomarkers for early diagnosis of UC could be instrumental in enhancing the early detection of CRC and mitigating the poor progression of UC to CAC.

Our cross-analysis of gene expression profiling in whole blood and mucosal tissues of patients with UC revealed that among the hub genes, *LCN2*, *MMP9*, *S100A9*, and *OLFM4* were selected as key hub genes and were consistently up-regulated in both blood and colonic mucosa (Figure 3). Notably, the gene expression regulatory regions of LCN2, S100A9, and OLFM4 were significantly hypo-methylated in the colonic mucosa of patients with UC, as revealed through DNA methylation profiling. This suggests that altered expression of these genes may be linked to epigenetic regulation. Echoing our results, recent research has seen an unprecedented surge in focusing on the role of genome-wide and gene-specific DNA methylation in driving the onset and progression of UC [44,45,46]. These studies underscore the growing recognition of epigenetic regulation as a critical player in disease pathogenesis. Notably, the hypo-methylation of specific genes, such as ZBTB7B (zinc finger and BTB domain containing 7B) and NLRP3, has been shown to correlate proportionally with the up-regulation of these genes, their corresponding proteins, and colonic inflammation, all of which are associated with the exacerbation of UC [45,46].

LCN2, also known as oncogene 24p3 or neutrophil gelatinase-associated lipocalin (NGAL), is primarily involved in innate immune responses and is predominantly expressed in the epithelial cells of the alimentary tract and neutrophils [47,48]. It is mainly synthesized and secreted when bacterial infections stimulate toll-like receptors (TLRs) in immune cells. It acts as an antimicrobial peptide by inhibiting bacterial growth through the sequestration of iron-containing siderophores [49]. Previous studies have reported that LCN2 is up-regulated in the colonic mucosa [50], serum [51], and feces [52] of patients with UC compared to their levels in healthy controls. Additionally, it is recognized as a potential inducer that leads to tumorigenesis from colitis through the IL-6/STAT3 (signal transducer and activator of transcription 3)/NF-κB (nuclear factor κB) signaling pathway [53]. Several studies have shown that the HIF-1α (hypoxia-inducible factor-1α) pathway [54] and ALOX15 (arachidonic acid 15-lipoxygenase) axis [55] mediate colonic inflammation via LCN2. This occurs through M1Ø polarization and proinflammatory cytokine secretion [54] as well as ferroptosis-mediated colonic physical barrier dysfunction, which exacerbates colitis [55].

S100A9, also known as migration inhibitory factor-related protein (MRP)-14 or calgranulin B, plays a key role in cell cycle progression and differentiation. This is facilitated by its Ca^2^⁺-binding capability through two EF-hand motifs and inhibition of casein kinase activity [56], which aligns with our GO analysis results related to the MFs of DEGs (Figure 1E). S100A9 forms a heterodimer with S100A8 (calprotectin complex), which modulates myeloid cell functions via interactions with TLR4 [57] and RAGE (receptors for advanced glycation end products) [58], consistent with the findings of our GO analysis on the MFs of DEGs (Figure 1E). Interestingly, this complex including S100A8 and p-S100A9 contributes to inflammatory regulation [59] by influencing neutrophil and MØ accumulation and promoting cytokine production in MØ [60]. Recent studies have revealed elevated levels of S100A9 in the peripheral blood mononuclear cells (PBMCs) of patients with IBD [61] and the serum of experimental colitis rats [62] compared to that in controls. Moreover, the anti-inflammatory effects of tasquinimod and hederacoside C were demonstrated by their ability to alleviate colitis, primarily through the inhibition of S100A9 release from MØ in mice [63] and neutrophils in a rat colitis model [64], respectively. Pediatric UC is often characterized by a more aggressive disease course compared to adult-onset UC, frequently presenting as pancolitis at diagnosis, in contrast to the typically left-sided involvement seen in adults [65]. This extensive inflammation in children is associated with marked infiltration of innate immune cells including neutrophils and MØ [66]. Given that S100A9 is abundantly expressed by activated neutrophils, monocytes, and MØ as part of the calprotectin complex, this inflammatory profile explains the markedly elevated expression of S100A9 observed in pediatric UC. Moreover, from an immunological development standpoint, children rely more heavily on innate immune responses due to the relative immaturity of their adaptive immune system [67,68]. This age-associated immune profile predisposes pediatric patients to more vigorous innate inflammatory reactions in the gut mucosa, including amplified production of proinflammatory cytokines, such as IL-1β and IL-8 [68], which in turn further enhance neutrophil recruitment and S100A9 expression. Therefore, the convergence of more extensive colonic involvement, higher neutrophilic activity, and heightened innate immune reactivity likely underlies the disproportionately elevated levels of S100A9 in pediatric UC compared to adult disease.

Calprotectin complex functions as a damage-associated molecular pattern (DAMP) that may contribute to CAC development via the classical “inflammation–dysplasia–carcinoma” sequence in the inflammatory milieu of UC. Specifically, this heterodimer has been shown to promote macrophage infiltration into colonic tissue and activate the Akt1–Smad5–Id3 signaling axis, ultimately facilitating tumor formation in the context of chronic colitis [69,70,71]. Supporting this mechanism, recent studies have highlighted the pivotal role of S100A9 in carcinogenesis, associating it with the dysregulated differentiation of myeloid cells within the tumor stroma and its involvement in the progression of leukemia [69]. Additionally, S100A9 is often elevated in various cancers including breast, prostate, colorectal, and lung cancers [72]. This over-expression may result from MØ-derived S100A9 contributing to the tumor microenvironment (TME), where it promotes chronic inflammation that facilitates tumor growth and metastasis [73]. Consequently, peptides [74] and antibodies [75] that specifically target S100A9 in the colon have shown efficacy in mouse models, reducing both colitis and CAC by inhibiting their interactions with TLR4 and RAGE.

OLFM4, often termed GW112 or hGC-1 (human G-CSF-stimulated clone 1), is a glycoprotein that is strongly expressed in the small intestine, colon, and prostate [76]. Emerging evidence indicates that OLFM4 is significantly up-regulated in the inflamed colonic epithelium of patients with active UC [77], with elevated levels of both OLFM4 and its secreted form in the colonic mucosal tissue and mucus of patients with UC [78]. This up-regulation may exacerbate UC by binding to the tissue-specific antimicrobial peptides (AMPs) and human β-defensins (HBD), thereby reducing their antimicrobial activities. Interestingly, a study showed that the OLFM4-metadherin (MTDH) complex up-regulates p38/RAR-related orphan receptor γt (RORγt) signaling, leading to the activation of IL-22^+^ ILC3 (group 3 innate lymphoid cells) during intestinal inflammation in humans and mice [79]. In carcinogenesis, OLFM4 shows a pattern of elevated expression during the early stages of CRC, which gradually declines and may become entirely absent in the advanced stages of the disease [80,81]. Additionally, in CAC, OLFM4 deficiency prevents disease progression by abrogating the recruitment of polymorphonuclear myeloid-derived suppressor cells [82].

From a practical clinical perspective, our identification of S100A9 as a blood-based biomarker holds promising implications. This suggests that hypo-methylation-driven up-regulation of key genes in colonic tissues, such as S100A9, may result in elevated blood expression, supporting their use in noninvasive diagnostic strategies. Noninvasive blood tests measuring circulating levels of S100A9 could significantly enhance early diagnosis, monitoring of disease activity, and risk stratification for patients with UC at risk of progression to CAC. For instance, routine blood screening for elevated S100A9 levels could be integrated into clinical practice, particularly benefiting pediatric and adolescent patient groups who require less invasive diagnostic methods. Furthermore, targeted therapeutic approaches, including specific antibodies or small-molecule inhibitors that disrupt S100A9 interactions with its receptors (such as TLR4 and RAGE), may provide novel precision medicine strategies to prevent chronic inflammation and subsequent tumorigenesis in high-risk patients. Nevertheless, rigorous clinical trials and validation studies remain essential to translate these promising preclinical findings into clinically actionable strategies.

This study has some limitations. Initially, our analysis primarily relied on GEO2R-based differential expression analysis and group comparisons using classical statistical tests, such as ANOVA and Tukey’s test. However, we acknowledge that these traditional approaches may not fully account for the underlying heterogeneity present in transcriptomic datasets, particularly in disease contexts like cancer and inflammatory conditions. To address these concerns and enhance the statistical rigor of our study, we implemented a LASSO-based variable selection strategy with 5-fold cross-validation. This approach enabled us to identify genes that most robustly distinguish UC from control samples, and notably, all four of our originally proposed key hub genes (*LCN2*, *MMP9*, *S100A9*, and *OLFM4*) were selected under this model. These findings reinforce the robustness of our hub gene selection from both a network and statistical standpoint. Furthermore, the GSE177044 dataset consisted of blood samples, whereas the validation datasets were derived from colonic biopsies. Although our primary dataset was derived from whole blood and the validation datasets from colonic biopsies, we observed that key hub genes up-regulated in colon tissue also showed elevated levels in blood, supporting their potential as systemic biomarkers reflective of colonic inflammation. This discrepancy in sample types could introduce variability in gene expression profiles. Nevertheless, the observed up-regulation of *LCN2*, *S100A9*, and *OLFM4* in blood may reflect underlying hypo-methylation and transcriptional activation in colonic tissue, supporting their potential clinical application as minimally invasive blood-based biomarkers for early UC diagnosis. Lastly, our findings lacked experimental validation. Although bioinformatics analysis identified S100A9 as a potential biomarker for early diagnosis and progression to CAC, future research incorporating comprehensive clinical annotations and experimental validations is warranted to fully elucidate its precise clinical implications and underlying mechanisms.

## 4. Materials and Methods

### 4.1. Next-Generation Sequencing (NGS) Dataset

The Gene Expression Omnibus (GEO), hosted by the National Center for Biotechnology Information (NCBI), is an openly accessible repository (https://www.ncbi.nlm.nih.gov/geo, accessed on 21 August 2024). In the present study, we conducted an extensive search using keywords, such as ”Inflammatory bowel disease”, ”IBD”, ”Ulcerative colitis”, and ”UC”, which led to the identification of the gene expression dataset GSE177044. This dataset, comprising 481 whole blood samples from patients with UC and 311 healthy controls, is accessible for further analyses through GEO2R (https://www.ncbi.nlm.nih.gov/geo/info/geo2r.html, accessed on 21 August 2024) [83]. All samples were normalized using the GEO2R tool. The clinical characteristics of the patients enrolled in this study are presented in Table 2. Only age and sex were available as clinical metadata, since the dataset originated from a public database that lacked further clinical information.

### 4.2. Identification of DEGs

We used GEO2R to perform a DEGs analysis of whole blood samples from patients with UC and healthy controls. DEGs were identified from the GSE177044 dataset by applying stringent criteria: adjusted *p*-values below 0.05 (calculated using the Benjamini and Hochberg false discovery rate [FDR] correction) and an absolute log_2_ fold change (|log_2_FC|) greater than 1.

### 4.3. GO and KEGG Pathway Analysis of DEGs

To achieve comprehensive gene annotation and functional enrichment analysis, we employed the Database for Annotation, Visualization, and Integrated Discovery (DAVID) web server (https://david.ncifcrf.gov) [84]. Using DAVID version 7.0, we performed GO and KEGG pathway analyses to uncover critical biological processes and pathways associated with the identified DEGs.

### 4.4. PPI Network Build Up for Hub Genes Detection

The STRING database (https://string-db.org), a comprehensive online resource for precomputed protein association networks [85], was used to analyze PPI networks. Specifically, STRING version 12.0 was used to detect hub genes among the DEGs, focusing on both up-regulated and down-regulated DEGs.

### 4.5. Validation Using Colon Mucosa Samples of Patients with UC

To explore the potential of blood-derived hub genes as biomarkers of UC, we performed a cross-analysis of three datasets: GSE59071 (97 inflamed colonic mucosa samples from patients with UC and 11 normal control samples), GSE66407 (161 biopsies from patients with UC and 99 normal control samples), and GSE87466 (87 colon tissue biopsies from patients with UC and 21 normal control samples). This analysis was conducted in conjunction with hub genes identification to select the key hub genes. Furthermore, to determine whether key hub genes were present in the DEGs of each dataset, we performed DEGs analysis for each dataset individually.

### 4.6. Variable Selection via Using LASSO

To validate the robustness of our hub gene selection, we further performed variable selection using LASSO. Log-transformed expression data (log_2_(count + 1)) from the GSE177044 dataset were standardized prior to modeling. We employed Logistic Regression from the Scikit-learn package (v1.2), applying a 5-fold cross-validation procedure to optimize model performance. Genes with nonzero coefficients were selected as informative features.

### 4.7. Analysis of DMRs of Key Hub Genes

Our aim was to uncover the influence of epigenetic mechanisms in driving the regulatory changes in the expression levels of key hub genes. To achieve this, we analyzed the GSE32149 dataset, which includes colonic mucosa samples from four patients with UC and ten healthy controls, to identify DMRs specific to key hub genes in patients with UC. Given that DNA methylation is generally associated with the repression of gene expression [86], we investigated the potential inverse correlation between DNA methylation and the expression of key hub genes.

### 4.8. Analysis of Age-Related Expression Patterns of Key Hub Genes in Patients with UC

We examined the GSE107597 dataset, which consisted of colon biopsy samples from four distinct groups: 41 pediatric and adolescent patients with UC, 46 adult patients with UC, 22 pediatric and adolescent non-lesional controls, and 22 adult non-lesional controls. Our objective was to determine whether epigenetic factors regulate changes in the expression levels of key hub genes. All samples were normalized using the GEO2R tool. The clinical characteristics of the samples are presented in Table 3. Clinical metadata were limited to age and sex only, as the dataset was derived from a public repository that did not include additional clinical parameters.

A summary of the datasets used in this study—including tissue type, sample size, age group, and available clinical metadata—is presented in Table 4. Detailed clinical metadata such as disease severity, treatment history, and disease duration were unavailable across most datasets, which limited our ability to perform stratified clinical analyses.

### 4.9. Analysis of the Effects of Key Hub Gene Expression on Cancer

To investigate whether the key hub genes associated with UC are potential candidates related to CAC, we analyzed the impact of these key hub genes on COAD, which represents the majority of colorectal cancers. We utilized the University of Alabama at Birmingham Cancer Data Analysis Portal (UALCAN, http://ualcan.path.uab.edu, accessed on 22 November 2024), incorporating clinical data from *The Cancer Genome Atlas* (TCGA) and the Clinical Proteomic Tumor Analysis Consortium (CPTAC) to examine the relative mRNA (COAD: *n* = 286; normal: *n* = 41) and protein (COAD: *n* = 97; normal: *n* = 100) expression, as well as DNA methylation levels (COAD: *n* = 313; normal: *n* = 37) in COAD tumor and control tissues [87]. To investigate the cell type-specific expression patterns of key hub genes in COAD, we used the GSE178341 scRNA-seq dataset. This dataset included 62 COAD tumors (*n* = 258,359 cells) and 36 normal colon tissue samples (*n* = 112,864 cells) accessed through The Single-Cell Portal (SCP) database (https://singlecell.broadinstitute.org/single_cell, accessed on 22 November 2024) [88]. Using unsupervised clustering, this dataset identified 88 sub-populations across seven major cell lineages, defining 204 gene expression programs that characterize the cellular and molecular landscape of COAD. This dataset enables precise analysis of gene expression dynamics within specific immune and epithelial compartments of the TME. Furthermore, we investigated the associations of key hub genes with various immune subtypes and molecular subtypes in patients with COAD (*n* = 441) utilizing an integrated repository portal for the tumor–immune system interactions database (TISIDB) (http://cis.hku.hk/TISIDB/index.php, accessed on 22 November 2024), an online platform for exploring tumor–immune system interactions [89]. Moreover, IHC-based protein expression data were sourced from the Human Protein Atlas (HPA) (https://www.proteinatlas.org, accessed on 22 November 2024) [90]. Finally, we used the Kaplan–Meier (KM) plot database (https://kmplot.com, accessed on 22 November 2024) to analyze key hub gene expression and evaluate their impact on survival outcomes in patients with COAD (OS: *n* = 1061; RFS: *n* = 1336; post-progression survival [PPS]: *n* = 331) [91].

### 4.10. Data Visualization and Statistical Analysis

Data visualization was performed using Hiplot (https://hiplot.cn, accessed on 26 July 2024) [92] and GraphPad Prism version 9 (GraphPad Software, San Diego, CA, USA). For statistical evaluation of the experimental results, ANOVA followed by Tukey’s multiple comparisons test for group comparisons was employed. A significance threshold was set at a *p*-value below 0.05.

## 5. Conclusions

Taken together, our study findings confirmed that the up-regulation of *LCN2*, *S100A9*, and *OLFM4* in the colons of patients with UC, driven by hypo-methylation, translates to increased concentrations of these biomarkers in the blood, suggesting their potential as blood-based diagnostic biomarkers. Furthermore, our analysis highlighted that, among these key hub genes, *S100A9* holds significant potential as a biomarker for the early diagnosis of UC, particularly in pediatric and adolescent patients. Clinically, S100A9 may also serve as a prognostic biomarker in precision medicine for patients with UC who are at risk of progressing to CAC.

## Figures and Tables

**Table 1:** Summary of LASSO-selected genes relevant to UC diagnosis.

Genes	LASSO Coefficient	log_2_FC	Adj. *p*-Value	Rank (of 113)
*LCN2*	−3.40228	1.42065	1.45 × 10^−37^	2
*MMP9*	−1.38405	1.45637	2.02 × 10^−51^	25
*S100A9*	−0.32635	1.32343	2.59 × 10^−85^	86
*OLFM4*	−0.05912	2.68168	1.26 × 10^−55^	110

**Table 2:** Clinical information of the samples within the GSE177044 dataset.

Characteristics	Ulcerative Colitis	Healthy Controls	*p*-Value
No. of patients	*n* = 481	*n* = 311	-
% of men/women	Men: 34.21% Women: 65.79%	Men: 67.11% Women: 32.89%	0.4538
Age (y), mean (SD)	41.09 ± 14.22	48.02 ± 12.14	< 0.001

**Table 3:** Clinical information of the samples within the GSE107597 dataset.

Characteristics	UC (Pediatric and Adolescent)	UC (Adult)	Control (Pediatric and Adolescent)	Control (Adult)
No. of patients	*n* = 41	*n* = 34	*n* = 22	*n* = 22
% of men/women	Men: 58.54% Women: 41.46%	Men: 47.06% Women: 52.94%	Men: 72.73% Women: 27.27%	Men: 54.55% Women: 45.45%
Age(y), mean (SD)	17.68 ± 2.27	37.29 ± 12.94	17.28 ± 2.25	37.09 ± 13.19

**Table 4:** Summary of publicly available datasets used in this study.

Datasets	Sample Type	UC Samples	Control Samples	Utilization
GSE177044	Whole blood	*n* = 481	*n* = 311	DEGs and hub gene selection
GSE59071	Colonic tissue	*n* = 97	*n* = 11	Hub genes validation based on shared expression patterns in both blood and tissue
GSE66407		*n* = 161	*n* = 99	
GSE87466		*n* = 87	*n* = 21	
GSE32149	Colonic tissue	*n* = 4	*n* = 10	Key hub genes selection
GSE107597	Colonic tissue	*n* = 75	*n = 44*	Age-related expression validation

## Data Availability

The data used in this study are available from the NCBI-GEO (https://www.ncbi.nlm.nih.gov/geo, accessed on 21 August 2024); UALCAN (http://ualcan.path.uab.edu, accessed on 22 November 2024); SCP (https://singlecell.broadinstitute.org/single_cell, accessed on 22 November 2024); TISIDB (http://cis.hku.hk/TISIDB/index.php, accessed on 22 November 2024); HPA (https://www.proteinatlas.org, accessed on 22 November 2024); and KM plot databases (https://kmplot.com databases, accessed on 22 November 2024). Data are also available upon reasonable requests to the corresponding author.
